# Dissecting Cellulitis of the Scalp: Linking Pathogenesis to Therapy

**DOI:** 10.3390/biomedicines14030570

**Published:** 2026-03-02

**Authors:** Mislav Mokos, Mirna Šitum, Ines Sjerobabski Masnec

**Affiliations:** 1 Department of Dermatology and Venereology, Sestre Milosrdnice University Hospital Center, 10000 Zagreb, Croatia; mirna.situm@kbcsm.hr (M.Š.); ines.sjerobabski.masnec@kbcsm.hr (I.S.M.); 2 School of Dental Medicine, University of Zagreb, 10000 Zagreb, Croatia; 3 Croatian Academy of Sciences and Arts, 10000 Zagreb, Croatia; 4 Faculty of Pharmacy and Biochemistry, University of Zagreb, 10000 Zagreb, Croatia

**Keywords:** dissecting cellulitis of the scalp, perifolliculitis capitis abscedens et suffodiens, hidradenitis suppurativa, follicular occlusion tetrad, biologic therapy, adalimumab, secukinumab, TNF-alpha inhibitors, IL-17 inhibitors, scarring alopecia

## Abstract

Dissecting cellulitis of the scalp (DCS) is a chronic, inflammatory follicular occlusion disorder characterized by painful nodules, abscesses, and sinus tracts that lead to scarring alopecia. The therapeutic goal is to limit disease progression and the extent of scarring. Although DCS is traditionally managed with systemic retinoids, antibiotics, and surgical interventions, therapeutic responses are variable and long-term remission remains challenging. Recent insights into the immunological overlap between DCS, hidradenitis suppurativa (HS), and other autoinflammatory follicular disorders have expanded therapeutic options, particularly with biologic agents targeting tumor necrosis factor alpha (TNF-α), interleukin (IL)-17, and IL-23 pathways, as well as Janus kinase (JAK) inhibitors. This review synthesizes the current evidence on medical, procedural, and emerging targeted therapies for DCS, incorporating data from case reports, case series, retrospective cohorts, and recent systematic reviews up to 2025. Special emphasis is placed on the evolving role of biologics and small-molecule inhibitors, which show growing promise for refractory or syndromic presentations. Current evidence supports a stepwise, phenotype-driven approach in which systemic retinoids remain first-line systemic therapy, while biologics represent a rational and increasingly evidence-supported option for moderate-to-severe, treatment-resistant, or syndromic disease. Further controlled studies are needed to define optimal sequencing, duration, and combination strategies for long-term management.

## 1. Introduction

Dissecting cellulitis of the scalp (DCS), also known as perifolliculitis capitis abscedens et suffodiens or Hoffman’s disease, is a rare, chronic inflammatory disorder classified among the neutrophilic cicatricial alopecias [1]. It predominantly affects young adult men, especially those with darker skin phototypes, and is characterized by painful nodules, abscesses, sinus tracts, and progressive scarring alopecia that can lead to substantial psychosocial morbidity. An early diagnostic clue, often preceding prominent inflammation, is the presence of comedones on the scalp, reflecting early follicular occlusion [2,3,4]. Trichoscopy can further support diagnosis by demonstrating follicular pustules, yellow crusts, keratotic plugs, and structureless yellow areas over erythematous, boggy alopecic plaques [5]. DCS usually follows a chronic, relapsing course that may persist for years. While spontaneous remissions have been described, this is unpredictable and does not reverse established scarring [6,7]. Moreover, there are no robust longitudinal data quantifying the likelihood of truly self-limiting disease.

DCS frequently occurs within the follicular occlusion tetrad (FOT), alongside hidradenitis suppurativa (HS), acne conglobata (AC), and pilonidal disease [8]. These conditions share a core pathogenic sequence (follicular hyperkeratosis, occlusion, rupture, and secondary neutrophilic inflammation) resulting in deep tissue destruction and scarring [9]. Increasing evidence highlights significant immunologic overlap between DCS and HS, including elevated TNF-α, IL-1β, IL-17, and IL-23 expression in lesional skin, supporting the concept of a systemic autoinflammatory component rather than a purely localized scalp disorder [8,9,10,11].

Management of DCS remains challenging. Conventional therapies, including systemic antibiotics, isotretinoin, corticosteroids, and surgical interventions, usually provide only partial or temporary benefit, with high relapse rates upon discontinuation [2,3,9,12,13,14,15,16]. These limitations have prompted growing interest in targeted immunomodulatory therapies. Biologic agents approved for HS, notably TNF-α inhibitors, have shown encouraging results in DCS, especially in syndromic or treatment-refractory cases [1,2,3,4,12,17,18]. More recently, IL-17 and IL-23 pathway inhibitors have arisen as promising options, reflecting evolving insights into the cytokine networks that sustain chronic inflammation in FOT disorders [12,19,20,21,22,23].

While several systematic and narrative reviews have covered DCS treatments, the field has changed rapidly in recent years, particularly with the introduction of targeted biologics and small-molecule therapies [2,3,4,24,25,26,27]. The increased use of cytokine-pathway inhibitors and intracellular signal transduction blockers has brought important treatment options that earlier reviews did not fully address. Because of these changes, an updated review is needed to incorporate the latest evidence, place these advances in a practical clinical context, and connect new molecular insights into follicular rupture-driven immune activation and cytokine-network dysregulation with treatment strategies.

In this review, we examine current medical, procedural, and targeted immunologic therapies for DCS, drawing on case reports, case series, retrospective cohorts, and recent systematic reviews up to December 2025.

The aim of this review is to synthesize current evidence on the pathobiology and management of DCS, with specific emphasis on linking molecular and immune mechanisms to therapeutic targets. We further summarize reported outcomes of medical and procedural interventions and provide a practical, evidence-informed approach to treatment selection and sequencing.

## 2. Methods

We performed a narrative literature review to identify publications reporting on the epidemiology, pathogenesis, and treatment of DCS. A comprehensive search of PubMed/MEDLINE, Embase, Web of Science, Scopus, and Google Scholar was conducted from database commencement to December 2025. Search terms included combinations of the following keywords and Medical Subject Headings (MeSH): “dissecting cellulitis of the scalp,” “perifolliculitis capitis abscedens et suffodiens,” “Hoffman’s disease,” “follicular occlusion tetrad,” “hidradenitis suppurativa,” “acne conglobata,” “pilonidal sinus,” “therapy,” “treatment,” “biologic therapy,” “tumor necrosis factor inhibitors,” “interleukin-17,” “interleukin-23,” “Janus kinase inhibitors,” “retinoids,” “antibiotics,” “photodynamic therapy,” “laser,” and “surgery.” Reference lists of relevant reviews and case series were additionally screened to identify further reports. We included case reports, case series, retrospective cohorts, clinical trials, and systematic reviews that described clinical features, therapeutic interventions, or outcomes in patients with DCS. Articles not available in English and publications lacking primary clinical data (e.g., conference abstracts without full text) were excluded. Given the rarity of DCS and the heterogeneity of available evidence, no formal quality assessment or meta-analysis was performed.

## 3. Pathogenesis of DCS

**Figure 1:**
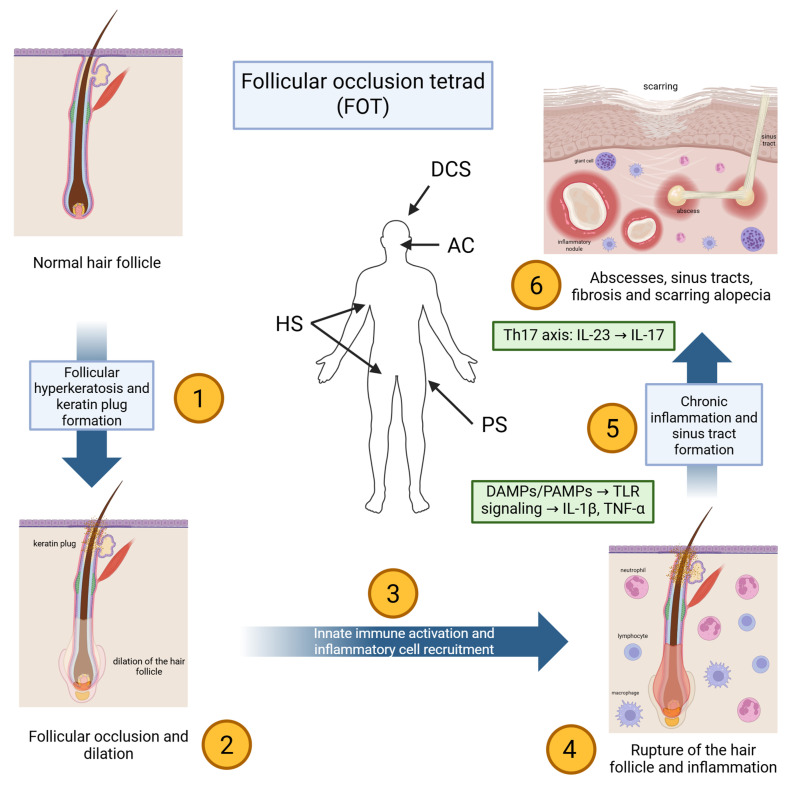
Pathogenesis of the follicular occlusion tetrad. This illustration shows the essentials steps in the pathogenesis of the follicular occlusion tetrad. Abbreviations: AC, acne conglobata; DAMPs, damage-associated molecular patterns; DCS, dissecting cellulitis of the scalp; FOT, follicular occlusion tetrad; HS, hidradenitis suppurativa; IL, interleukin; PAMPs, pathogen-associated molecular patterns; PS, pilonidal sinus; Th17, T helper 17 cell; TLR, Toll-like receptors. Created in BioRender. Mokos, M. (2025). https://BioRender.com/7y50gcp, accessed on 1 February 2026.

The pathogenesis of DCS is multifactorial, involving a complex interplay between follicular occlusion, microbial factors, immune dysregulation, and cytokine-driven inflammation (Figure 1) [8,28,29,30,31,32,33,34,35,36]. Even though the condition remains incompletely understood, increasing insights into its pathophysiology have revealed striking parallels with HS, which would imply shared etiopathogenetic pathways and therapeutic targets [4,28,31,32,33,34,35,36,37,38].

### 3.1. Initiation: Follicular Occlusion and Rupture

The pathogenic sequence of DCS begins with follicular hyperkeratosis, which obstructs the pilosebaceous unit at the infundibulum [1,8,9]. This blockage causes keratin and sebum to build up in the follicle, leading to gradual dilation [30,38,39]. Eventually, the dilated follicle ruptures, releasing keratin, sebum, bacteria, and damaged epithelial cells into the surrounding dermis [8,10,40]. This rupture induces an intense local immune response, initiating the characteristic inflammatory cascade of the disease [28].

Following rupture, dermal exposure to follicular contents introduces both endogenous danger-associated molecular patterns (DAMPs) and microbial-associated signals that can be sensed by pattern-recognition pathways in keratinocytes and resident immune cells (e.g., Toll-like receptor (TLR) signaling). This early innate immune sensing promotes rapid production of pro-inflammatory mediators and chemotactic gradients, favoring neutrophil recruitment and abscess formation [25,40,41,42,43,44,45,46]. In parallel, innate immune activation provides a mechanistic framework for the elevated IL-1β and downstream cytokine networks reported in DCS lesions and serum [28,47,48].

### 3.2. Innate Immune Activation and Neutrophilic Inflammation

Histopathological analysis of early lesions demonstrates a dense infiltrate composed primarily of neutrophils, along with lymphocytes, macrophages, plasma cells, and histiocytes [40,42,43]. The ensuing inflammation leads to the development of deep-seated abscesses and interconnected sinus tracts within the dermis and subcutis [44,46]. Over time, chronic inflammation promotes tissue destruction, granulation tissue formation, and ultimately, fibrotic scarring that manifests clinically as cicatricial alopecia [25,45].

### 3.3. Cytokine Networks and Th17-Associated Inflammation

DCS is classified as one component of the FOT, alongside HS, AC, and pilonidal sinus [8]. All four conditions share a core pathophysiological mechanism centered on follicular occlusion followed by follicular rupture and secondary inflammation [49,50,51]. These syndromes often co-occur in affected individuals, further supporting the concept of a common etiopathogenic substrate. Notably, the significant clinical and histological overlap between DCS and HS has led to increasing recognition of their shared immunopathology, particularly in the cytokine and cellular profiles of the lesions [8,38,52,53].

Recent advances in immunodermatology have elucidated the central role of pro-inflammatory cytokines in the pathogenesis of DCS. Elevated levels of TNF-α, IL-1β, IL-17, and IL-23 have been observed in lesional tissue and serum, which is practically identical to the findings in HS [28,47,48]. These cytokines collectively drive Th17 polarization, sustaining chronic inflammation within the skin [54]. TNF-α, a pleiotropic cytokine, contributes to keratinocyte activation, endothelial cell adhesion molecule expression, and further leukocyte recruitment [29,33,37]. IL-17 and IL-23, key components of the Th17 axis, promote neutrophil chemotaxis and enhance the release of antimicrobial peptides and pro-inflammatory mediators from epithelial cells [4,19,21,54,55].

Additionally, the IL-23/IL-17 axis has been shown to contribute to the chronicity and severity of inflammatory skin diseases through amplification of the local immune response [4,19,21]. IL-17 binds to its receptor (IL-17RA/RC) on keratinocytes and fibroblasts, inducing the release of further cytokines and matrix metalloproteinases, exacerbating tissue damage [37,54,55]. IL-23, in turn, promotes the survival and expansion of IL-17-producing T cells, further perpetuating this inflammatory loop [12,22].

### 3.4. Intracellular Signaling: JAK/STAT as a Convergent Node

Emerging evidence also underscores the importance of the Janus Kinase (JAK)/Signal Transducer and Activator of Transcription (STAT) pathway in DCS pathogenesis [4,56,57,58,59,60]. Transcriptomic studies, particularly in HS, have shown upregulation of JAKs and their downstream effectors. This is quite important since it suggests a significant role of JAKs in cytokine signal transduction, cellular proliferation, and immune cell activation in HS [57]. Inflammatory cytokines such as IL-6 and IFN-γ utilize this pathway to propagate signals that sustain inflammation and inhibit regulatory T-cell function [4,17]. Inhibition of the JAK/STAT axis has shown promise in HS and may represent a novel therapeutic modality in DCS as well [56,57,58,59,60].

### 3.5. Microbial Dysbiosis and Secondary Colonization as Amplifiers

Although DCS is not primarily an infectious disease, secondary bacterial colonization is frequently observed. Pathogens such as Staphylococcus aureus, Staphylococcus epidermidis, and Propionibacterium acnes are commonly isolated from lesional cultures [13,16,61]. These microbes probably do not trigger the disease, but they certainly can exacerbate the inflammatory response through the activation of TLRs on keratinocytes and immune cells. This then further enhances cytokine production and neutrophilic infiltration [3,16,62]. However, the failure of antimicrobial therapies to produce durable remissions highlights the importance of autoinflammatory mechanisms over infection per se [28].

### 3.6. Host Modifiers: Genetic and Hormonal Factors

While comprehensive genetic studies are limited due to the rarity of the disease, familial clustering and co-segregation with HS suggest a genetic predisposition involving innate immune regulation and keratinocyte biology [35,63]. Hormonal factors could also play an important role, given the disease’s predilection for post-pubertal males and the anecdotal therapeutic effectiveness of anti-androgens [1,16,35,63,64]. However, the specific genetic variants and hormonal pathways contributing to DCS still remain poorly defined.

All in all, current evidence suggests that follicular occlusion and rupture initiate innate immune activation and neutrophil-driven inflammation. This process is maintained by interconnected cytokine networks, especially TNF-α, IL-1β, and the IL-23/IL-17 axis. JAK/STAT acts as a common intracellular signaling point for several inflammatory mediators [4,28,47,48,54,55,56,57,58,59,60]. This understanding supports targeted immunomodulatory treatments for refractory disease and underpins the pathway-to-therapy mapping shown in Table 1.

## 4. Topical Therapy

Although topical therapies have been explored in DCS, the evidence base is limited and heterogeneous. Most reports are anecdotal (single case reports), while a smaller subset includes small case series or retrospective cohorts that may be more reproducible but still low quality. For instance, in an anecdotal single case report, a 14-year-old boy experienced disease improvement with the application of 15% resorcinol cream twice per day [66]. Similarly, in a single case report, a 20-year-old male showed symptomatic relief when treated with a combination of topical isotretinoin and clindamycin gels [67].

Furthermore, topical retinoids have limited evidence in HS as well, with only a few anecdotal case reports showing potential benefit when combined with other treatments [68]. Their use in HS remains largely experimental, and efficacy data are minimal [68].

Local application of diclofenac sodium gel has been reported in a small case series with symptomatic improvement in mild DCS [69]. Three young men (ages 17–24) with mild DCS were treated with 1% diclofenac sodium gel applied twice daily for three months [69]. All experienced notable reduction in pain, inflammation, and scalp nodules size. Moreover, there was even evidence of hair regrowth [69]. While encouraging, these findings require replication in larger cohorts.

In addition, limited but more reproducible evidence comes from retrospective review that evaluated the outcomes of topical antibiotic use in 11 patients. Only three individuals achieved partial clinical responses, while four developed local adverse reactions, including erythema and pruritus [26].

In cases where conventional treatments offer limited benefit, the alternative forms of local therapies may be an option [3]. Intralesional corticosteroid injections, though rarely reported, have demonstrated transient efficacy [7,26]. In individual case reports, patients showed partial resolution of lesions following steroid administration. However, outcomes were inconsistent, and treatment was sometimes complicated by adverse effects (AEs) such as localized skin atrophy or eventual disease recurrence after initial improvement [7,26].

Another emerging approach is intracavitary foam sclerotherapy, reported in a small case series, in which sclerosant agents were injected directly into sinus tracts or abscess cavities [70]. Preliminary results suggest that this method may promote lesion flattening and fibrotic remodeling without notable procedural complications, suggesting its potential as a minimally invasive adjunct in select patients [70].

Additionally, mechanical compression therapy has been described in an unconventional yet promising single case report, where a patient applied continuous pressure using a self-fabricated dressing system [71]. Over several months, this technique was associated with a marked reduction in lesion volume and evidence of terminal hair regrowth [71].

Topical and localized treatments are mainly backed by anecdotal evidence and should be seen as add-on options, not as main therapies that change the course of disease. Overall, most topical approaches (e.g., resorcinol, topical retinoid/antibiotic combinations, intralesional steroids, and compression) are anecdotal, whereas diclofenac gel (small case series) and topical antibiotics (retrospective cohort) represent the few approaches with limited observational reproducibility. When these treatments help, it is likely due to reducing local inflammation, lowering microbial load, or helping with drainage, rather than reversing the main process that starts the disease. The biggest challenge is the lack of standard outcomes and controlled studies. Future research should identify which local treatments add real value when used with systemic therapy and whether any can reliably improve symptoms or prevent relapse in early-stage disease.

## 5. Systemic Retinoids

As explained in Section 3, DCS begins with follicular hyperkeratosis and blockage, which can lead to follicular swelling and rupture. This is followed by activation of the innate immune system and inflammation driven by cytokines. Systemic retinoids, which help normalize follicular keratinization and reduce blockage, are important because they may help prevent rupture and the resulting inflammation (Table 1). They have been widely used in the management of DCS (Table 2) [2,24,25,72,73,74]. Among these agents, isotretinoin remains the most extensively studied and is considered the primary systemic retinoid option for DCS [72,73,74,75].

A systematic review of 57 studies identified isotretinoin as the most frequently reported therapy, used in approximately 53% of all cases, with 54% achieving significant improvement and a 19% relapse rate [24]. Another retrospective cohort of 51 patients likewise found isotretinoin to be the most effective agent, with 33 of 35 patients treated at 0.5–0.8 mg/kg/day achieving full remission within three months, although relapse after discontinuation was common [30].

Across published reports, isotretinoin dosing typically ranges from 0.25 to 1 mg/kg/day, and treatment duration strongly influences response duration. Many patients experience rapid improvement within 2–4 months, particularly with higher-dose regimens [13,24,27,30,40,74,75,76,77]. Case series and retrospective reviews have documented durable remission in long-standing, treatment-resistant DCS with doses of 0.75–1 mg/kg/day continued for several months [30,40,75,77]. However, relapse rates remain substantial, ranging from 20–40% in studies where isotretinoin was withdrawn before achieving adequate cumulative dosing or full inflammatory suppression [13,24,27,30,40,74,75,76,77].

Lower-dose regimens (e.g., 0.25–0.5 mg/kg/day or ~10 mg/day) have been increasingly used to improve tolerability. Evidence suggests that low-dose isotretinoin may still yield marked clinical improvement, particularly when combined with short courses of corticosteroids, topical antiseptics, or adjunctive antibiotics [73,78,79,80]. A multicenter study of 72 adults treated to a cumulative dose of 120–150 mg/kg reported a 90.3% positive response rate, underscoring that even reduced doses may be effective when administered for adequate duration [79]. Pediatric experience remains limited but includes reports of excellent responses with low-dose regimens [80].

Additional retrospective analyses reinforce isotretinoin’s overall value: in one study, 12 of 16 patients experienced partial or complete remission, while a meta-analysis of five studies reported 90% overall efficacy but a 24% relapse rate [13,40]. Taken together, these data support isotretinoin as the best-supported first-line systemic treatment for moderate-to-severe DCS, while also highlighting the need for long-term monitoring and individualized treatment duration.

Overall, research on retinoids shows they consistently help during the inflammatory stage of DCS, which aligns with the key roles of follicular occlusion and altered follicular keratinization at the initial phase of the disease. However, frequent relapses after treatment cessation suggest that keratinization modulation alone may not be enough to control immune activation once chronic inflammation and sinus tracts have formed. The findings are difficult to interpret due to differences in dosing, follow-up times, and other treatments. Future studies that use standard outcome measures and clear definitions of relapse would help determine the best dosing, tapering, and maintenance strategies.

**Table 2:** Systemic retinoid therapy in DCS.

Study Type	Cohort Size/Patient Details	Retinoid & Regimen	Clinical Outcomes	Follow-Up	AEs	Ref.
Systematic review	57 studies, mixed cases	Isotretinoin (varied regimens)	54% significant improvement; 19% relapse	Not specified	Not specified	[24]
Retrospective analysis	51 patients, 35 on isotretinoin (0.5–0.8 mg/kg/day)	Isotretinoin	Full remission in 33/35 within 3 months; frequent relapse post-discontinuation	Mean 6.7 months	Not specified	[30]
Case report	Single male patient	Isotretinoin (long-term)	Resolution after antibiotics failed	2 years	None reported	[76]
Case report	38-year-old male	Isotretinoin 0.7 mg/kg/day × 6 months	Adequate control after 2 months; maintained on 6 months	6 months	None reported	[27]
Case series	3 adults, long-standing DCS	Isotretinoin 0.75–1 mg/kg/day	Durable remission	Up to 2.5 years	Not specified	[75]
Case report	25-year-old man	Isotretinoin × 1 year	Near-complete remission, no relapse at 6 months post-treatment	18 months total	None reported	[74]
Case report	18-year-old male	Low-dose isotretinoin 0.27 mg/kg/day × 4 months	Near-complete remission	7 months	None reported	[73]
Case report	Young male	Low-dose isotretinoin 10 mg/d (~0.2 mg/kg) + corticosteroids, doxycycline, clobetasol	Marked reduction in nodules; halted progression	Several months	Not specified	[78]
Retrospective multicenter study	72 adults	Low-dose isotretinoin 0.25–0.5 mg/kg/day until 120–150 mg/kg cumulative	90.3% positive response; improved across all stages	Not specified	Not specified	[79]
Case report	Pediatric patient (young girl)	Low-dose isotretinoin	Excellent response	Not specified	None reported	[80]
Multicenter retrospective	21 patients, 8 treated	Isotretinoin 30 mg/day	7/8 with significant reduction in activity	Not specified	Not specified	[77]
Retrospective review	16 patients	Isotretinoin (varied)	12/16 full or partial remission; 2 recurrences with low cumulative dose	Not specified	Not specified	[40]
Meta-analysis	5 studies	Isotretinoin	Overall efficacy 90%; recurrence 24%	Not specified	Not specified	[13]
Case report	15-year-old male with KID syndrome + DCS	Alitretinoin 10–20 mg/day × 5 months	Marked improvement; sustained benefit	5 months	No significant AEs	[81]
Case report	37-year-old female with KID syndrome + DCS	Alitretinoin 10–30 mg/day × 5.5 months	Near-complete resolution	5.5 months	Not specified	[82]
Case report	32-year-old male	Acitretin 25 mg/day	Noticeable improvement of nodules and draining lesions	Limited follow-up	Not specified	[83]

Abbreviations: AE, adverse event; DCS, dissecting cellulitis of the scalp; KID, keratitis–ichthyosis–deafness.

### Clinical Perspective

Isotretinoin remains the preferred first-line systemic option for predominantly inflammatory DCS. Because relapse after discontinuation is common, management often requires longer courses and/or combination therapy, with escalation to biologics for persistent, refractory, or syndromic disease. Alternative retinoids can be considered when isotretinoin is contraindicated or poorly tolerated.

## 6. Oral Antibiotic Therapy

DCS is not mainly an infectious disease, but changes in the normal microbes and secondary colonization can increase inflammation by triggering innate immune pathways, which keeps the neutrophil-rich inflammation going (Table 1). Antibiotics are used to lower bacterial levels when needed and also to help reduce inflammation. Still, since the main problem is immune dysregulation driven by cytokines, using only antibiotics often does not fully or lastingly control the disease [3,75]. Systemic antibiotics are commonly used in the management of DCS, and they are often employed as first-line systemic agents or as adjuncts to other therapies (Table 3).

Tetracyclines and related agents represent the most frequently reported antibiotic class in DCS treatment [2,26,30,78,84]. In a retrospective analysis, lymecycline 300 mg daily for three months led to clinical improvement in 9 of 10 patients, illustrating a relatively high initial response rate [84]. Doxycycline has also been evaluated in both prospective and retrospective settings. A small prospective trial of doxycycline monotherapy over three months showed favorable improvement in all seven participants, but no patient achieved complete remission, emphasizing the partial and temporary nature of antibiotic benefit [2]. In a larger series involving multiple regimens, doxycycline, dapsone, and rifampicin–clindamycin combinations produced partial responses in most patients, with only a minority achieving full hair regrowth and several individuals developing gastrointestinal AEs [26].

Beyond tetracyclines, a variety of other antibiotic regimens have been described in case reports and small series. Quinolones, including ciprofloxacin, have been associated with rapid symptomatic improvement in treatment-refractory DCS following failure of multiple prior therapies, including isotretinoin and combination antibiotics [85,86]. Rifampicin should not be used as monotherapy because resistance can emerge rapidly; when used in DCS, it should be prescribed only in combination regimens (most commonly with clindamycin). Reports describing rifampicin monotherapy-associated improvement should therefore be interpreted cautiously, particularly when concomitant agents (e.g., zinc or systemic corticosteroids) were used, which limits attribution of efficacy [26,87]. Oral clindamycin alone has shown satisfactory improvement in single-patient reports, but evidence remains anecdotal [78,88].

**Table 3:** Systemic antibiotic therapy in DCS.

Study Type	Cohort Size/Patient Details	Antibiotic Regimen	Clinical Outcomes	AEs	Ref.
Retrospective analysis	10 patients	Lymecycline 300 mg daily × 3 months	9/10 improved	Not specified	[84]
Case report	Single patient	Oral clindamycin	Satisfactory improvement	Not specified	[88]
Case report	28-year-old male, refractory DCS	Quinolone (after failure of doxycycline, zinc, dapsone + rifampicin, isotretinoin)	Clinical improvement	Not specified	[85]
Case report	Single patient, recurrent DCS	Ciprofloxacin	Successful treatment; rapid, well tolerated	Not specified	[86]
Case report	22-year-old male, refractory	Rifampicin × 6 months + zinc cream + systemic steroids	Near-complete regression	Not specified	[87]
Retrospective analysis	14 patients	6 doxycycline, 4 dapsone, 4 rifampicin + clindamycin	3 had full hair regrowth; majority partial improvement	4 had GI side effects	[26]
Prospective trial	7 patients	Doxycycline monotherapy × 3 months	Favorable improvement; no full remission	Not specified	[2]
Retrospective study	40 patients	Various antibiotic protocols	Moderate improvement; frequent relapse after discontinuation	Not specified	[30]
Case report	13-year-old patient	Several antibiotics (doxycycline, clindamycin)	No remission; disease persisted until isotretinoin, steroids, biologics	Not specified	[78]

Abbreviations: AE, adverse effect/event; DCS, dissecting cellulitis of the scalp; GI, gastrointestinal.

Despite these observations, durability of antibiotic responses is limited. In a retrospective series of 40 patients treated with various systemic antibiotic protocols, most experienced only moderate, transient improvement, with frequent relapse shortly after discontinuation [30]. Similarly, repeated courses of doxycycline and clindamycin failed to control inflammatory nodules and pustules in a pediatric case, and sustained disease control was only achieved after escalation to isotretinoin, systemic corticosteroids, and biologic therapy [78].

Antibiotics usually provide the most reliable short-term relief from pain, drainage, and pustules, which supports the idea that bacteria and neutrophil-driven inflammation play a role in active lesions. Still, the frequent recurrence of symptoms after antibiotic cessation suggests that these drugs rarely address the main causes of DCS, such as follicular occlusion and the cytokine networks underlying chronic inflammation. Since most studies have small sample sizes, mixed treatments, and short follow-up, future research should focus on using standard outcome measures, clearly separating anti-inflammatory from antimicrobial effects, and comparing common regimens, including how long they are used and in what combinations, as a bridge to longer-term disease control.

### Clinical Perspective

Systemic antibiotics are best used as short-term initial/bridging or adjunctive therapy (including treatment of suspected secondary infection), but durable remission with antibiotics alone is uncommon. Ongoing or moderate-to-severe disease should prompt transition to a broader strategy (retinoids, targeted/biologic therapy, and/or procedures based on phenotype and severity).

## 7. Biologic Therapy

Growing evidence shows that interconnected cytokine networks play a role in DCS, especially TNF-α signaling and Th17-related inflammation supported by the IL-23/IL-17 axis, which together offer a molecular basis for targeted immunomodulation (Table 1). By blocking these early inflammatory pathways, biologic therapies aim to lower leukocyte recruitment, keratinocyte activation, and the ongoing inflammation that leads to abscesses, sinus tracts, and scarring [3,4,12,19,21,89].

### 7.1. TNF-α Inhibitors

TNF-α inhibitors are the most commonly used biologic therapy for DCS and are the focus of much of the scientific literature on targeted immunomodulation for this condition (Table 4) [18,29,30,59,90,91,92,93,94,95,96]. Their use is supported by parallels between DCS and HS, including shared follicular occlusion mechanisms, increased TNF-α expression, and clinical overlap in syndromic cases [59,91,92]. The agents most frequently reported include infliximab, adalimumab, and, to a lesser extent, certolizumab pegol.

A large retrospective cohort of 26 patients with severe, treatment-refractory DCS evaluated infliximab or adalimumab as third-line therapy. Over a median follow-up of 19 months, patients experienced substantial reductions in inflammatory nodules, abscesses, pain scores, and DLQI, along with improvements in physician global assessment and patient satisfaction [90]. Despite these benefits, eight patients discontinued therapy, including two due to serious AEs, retrobulbar optic neuritis and hepatic cytolysis, highlighting the need for careful monitoring [90].

Several case reports and small case series provide additional evidence of TNF-α inhibitor efficacy. Multiple patients with DCS and concomitant HS demonstrated marked clinical improvement within weeks of initiating adalimumab, including reductions in drainage, tenderness, and lesion count, and in some cases partial hair regrowth [59,91,92]. Dose escalation from 40 mg to 80 mg every other week has been reported to maintain disease control in refractory cases [91]. Sustained benefit over 9–15 months has been described in individuals who previously failed oral antibiotics, isotretinoin, or combination systemic regimens [91,92].

Infliximab has also shown meaningful improvement in several reports, with reductions in pain, purulent drainage, and nodulocystic lesions, particularly in patients with co-existing HS [18,59,90]. However, infliximab treatment may obtain variable results, with at least one documented case showing minimal improvement after almost a year of therapy [30]. Notably, infliximab-associated optic neuritis has been observed in more than one report, underscoring a rare but important AE profile [18].

Beyond these larger agents, certolizumab pegol has been described in a single case involving a pregnant woman with active DCS. Treatment resulted in meaningful improvements in pain, erythema, and purulent discharge over four months, without maternal or fetal complications [96]. Although data remain extremely limited, this experience suggests that certolizumab may be considered in pregnancy when a TNF-α inhibitor is indicated.

Overall, TNF-α inhibitors show the most consistent clinical results among targeted treatments for DCS. This is supported by the largest retrospective cohort data and several case reports. These treatments tend to reduce inflammation, pain, and drainage, even in patients with severe or overlapping HS. This pattern matches the idea that TNF-driven inflammation plays a role in follicular occlusion syndromes. However, differences in patient responses, off-label dosing, and side effects that lead to stopping treatment make it hard to judge how long the benefits last or which patients will benefit most. The next important step is to collect prospective, multicenter data using standard clinical endpoints and safety measures, ideally along with biomarker sampling to help identify which inflammatory types are most likely to respond to TNF blockers.

#### Clinical Perspective

TNF-α inhibitors are the most evidence-supported biologic option for moderate-to-severe, refractory DCS, particularly with overlapping HS or syndromic disease. Given variable response and potential relapse after cessation, treatment should include appropriate safety screening/monitoring and a plan to switch class (e.g., to IL-17/IL-23-targeting agents) if response is inadequate, lost, or not tolerated.

### 7.2. IL-Targeting Biologics

In addition to TNF-α inhibitors, biologic agents that target IL pathways have been increasingly reported in the management of DCS. These include IL-17A inhibitors (secukinumab), IL-23 inhibitors (guselkumab, risankizumab, tildrakizumab), and agents targeting both IL-12 and IL-23 (ustekinumab) [12,19,20,21,22,23,65]. Their use is supported by growing evidence that the IL-23/IL-17 axis plays a central role in the immunopathogenesis of follicular occlusion disorders, including DCS and HS [12,19,20,21,22,23,65].

#### 7.2.1. Secukinumab

Secukinumab, an IL-17A inhibitor, is currently the most frequently reported IL-targeting biologic in DCS and has been associated with rapid and sometimes durable responses (Table 5) [19,20]. In one case, a 63-year-old man with long-standing, biopsy-proven, isolated DCS of the occipital scalp achieved near-complete remission after six weekly loading doses followed by monthly maintenance injections [19]. A marked reduction in nodules, abscesses, and suppuration has been noticed, with stabilization of disease activity at one year. As an AE, a transient eczema appeared early after the introduction of therapy but was successfully managed with topical treatment [19].

A second report described a young man with syndromic disease involving DCS, HS, AC, and pilonidal sinus, who had failed isotretinoin, antibiotics, and adalimumab [20]. Standard-dose secukinumab (300 mg weekly for five weeks, then monthly) resulted in gradual improvement of DCS and HS lesions, reduced pain and discharge, and better quality of life, without significant AEs [20].

#### 7.2.2. IL-23 and IL-12/23 Inhibitors

Experience with IL-23 inhibitors in DCS remains limited but promising. Guselkumab has been reported to induce a robust clinical response in a patient with complex disease involving DCS, HS, folliculitis, AC, and pyoderma gangrenosum after failure of adalimumab [21]. The patient experienced near-complete healing of scalp lesions and resolution of systemic symptoms, suggesting that selective IL-23 blockade may be effective in highly inflammatory, syndromic presentations [21].

Similarly, risankizumab has demonstrated favorable efficacy and safety in small numbers of patients with refractory DCS [22,65]. In two documented cases, treatment led to resolution of inflamed nodules, absence of new purulent lesions, and visible hair regrowth after several doses, with no serious AEs reported [22,65]. Tildrakizumab achieved significant clinical improvement in a patient with overlapping DCS, HS, and AC, including reduced pustular activity, scalp tenderness, and improved hair density (9). These reports collectively suggest a class effect of IL-23 inhibition in controlling inflammation and promoting partial hair recovery in DCS [12].

In contrast, ustekinumab, which targets both IL-12 and IL-23, has shown inconsistent benefit. In a case of a patient with inflammatory bowel disease and DCS-like lesions, there was no meaningful improvement after ustekinumab treatment [23]. This discrepancy suggests the heterogeneity of IL-pathway involvement across individuals and the need for personalized biologic selection.

Observational data indicate that approximately one-quarter to one-third of patients treated with IL-targeting biologics have comorbid immune-mediated skin disease, particularly HS and AC [12,21]. This reinforces the concept that IL-17/23-directed therapy may be especially valuable in syndromic follicular occlusion phenotypes.

So far, early clinical experience with IL-17 and IL-23 pathway inhibitors shows promise for certain patients, especially those with disease in multiple body areas or who have not responded to TNF-α inhibitors. This fits with new evidence that links Th17-axis cytokines and related inflammation to follicular occlusion disorders like DCS. Still, most of the current data comes from case reports and small studies that use different treatments and outcome measures, which makes it hard to compare results. Future research should focus on prospective studies with consistent clinical endpoints, including hair outcomes when relevant, clearer baseline staging, and biomarker assessments to see if a Th17-skewed inflammatory profile predicts better response.

#### 7.2.3. Clinical Perspective

IL-17 and IL-23-targeting biologics are reasonable next-line options for selected patients with refractory DCS, especially after TNF-α inhibitor failure or intolerance. Evidence remains limited to small observational reports; therefore, these agents are best used in highly selected cases, preferably in collaboration with centers experienced in complex follicular occlusion disorders and scarring alopecia.

## 8. JAK Inhibitors/Small-Molecule Targeted Therapies

JAK/STAT signaling acts as a central point inside cells for several cytokines linked to DCS. Blocking JAK is a strategy for difficult-to-treat disease and fits the framework shown in Table 1. The emerging role of JAK inhibitors in scarring alopecias has been highlighted in a recent narrative review, which noted early clinical success across several off-label applications [55].

Among the limited published reports of small-molecule targeted therapy used directly in DCS, upadacitinib, a selective JAK1 inhibitor, has been described in a case report. The authors described a patient with recalcitrant disease, who experienced marked improvement after initiation of upadacitinib, with reductions in pain, inflammatory nodules, and drainage [56]. This case supports the mechanistic rationale for targeting JAK1-dependent cytokine pathways in highly resistant DCS.

Yu et al. provided more clinical evidence for targeting the JAK pathway in DCS by describing a 15-year-old patient whose DCS did not respond to antibiotics or incision and drainage [1]. The patient first improved with adalimumab and isotretinoin, then started baricitinib (4 mg daily) as an additional treatment, later switching to a less frequent maintenance schedule. Over nine months, the patient’s lesions nearly cleared, inflammatory alopecia improved, and hair regrowth was seen. While lipid abnormalities occurred during the isotretinoin and adalimumab phase, no side effects specific to baricitinib were reported [1].

Further evidence comes from combination therapy reports. Tofacitinib, a JAK1/3 inhibitor, has been successfully used alongside ixekizumab in a patient with refractory DCS overlapping with features of severe HS. The combined regimen resulted in substantial improvement in inflammation and symptomatic burden [58]. Although the relative contribution of tofacitinib versus IL-17A blockade is not fully delineated, this case supports the potential benefit of integrating small-molecule inhibitors into biologic-based regimens in complex presentations.

Beyond combination regimens, Jin et al. described a difficult, long-term DCS case that was treated with abrocitinib (100 mg daily) after incision and drainage [97]. The patient reached clinical remission in 4 months and stayed in remission for a year. This case adds some, though still limited, clinical support for using JAK1-selective inhibition in highly refractory DCS [97].

Similarly, Al-Mamoori et al. reported a patient with refractory DCS who showed significant improvement on tofacitinib (10 mg once daily) [98]. The patient’s active inflammatory lesions went into remission by 9 weeks and remission was maintained for 6 months. No AEs or lab abnormalities were seen, but the authors noted that this use is off-label and needs careful monitoring [98].

Additional support arises from closely related research in HS. In two phase 2 studies, povorcitinib (INCB54707), another selective JAK1 inhibitor, produced transcriptomic and proteomic improvements in HS lesions, downregulating inflammatory pathways shared with DCS (including neutrophil chemotaxis, Th17-axis cytokines, and keratinocyte-derived mediators) [57]. Although these trials did not include DCS patients, they reinforce the theoretical basis for JAK inhibition in follicular occlusion disorders and provide early insight into safety and molecular response patterns.

Systemic JAK inhibitors carry class warnings for serious infections (including opportunistic infections), malignancy, major adverse cardiovascular events, and venous thromboembolism (VTE), based largely on safety signals observed in rheumatoid arthritis populations [99]. Regulatory agencies have therefore recommended that JAK inhibitors be used cautiously and generally after suitable alternatives, with avoidance or heightened caution in higher-risk groups (e.g., age ≥ 65 years, long-term current/past smokers, prior atherosclerotic cardiovascular disease, VTE risk factors, or prior malignancy) [100,101]. In contrast to typical RA cohorts, DCS patients are often younger. However, many have comorbid follicular occlusion disease (particularly HS) and related risk factors such as obesity, smoking, and metabolic syndrome, which may increase baseline cardiometabolic and thromboembolic risk [99,102]. In addition, active draining lesions and prior or concomitant immunosuppressive therapies (e.g., systemic retinoids and biologics) may increase infection risk [99,102]. Accordingly, if a JAK inhibitor is considered in refractory DCS, clinicians should emphasize careful patient selection, infection risk mitigation (including tuberculosis and viral hepatitis screening where appropriate), vaccination optimization (including zoster where eligible), and laboratory monitoring (complete blood count, liver enzymes, and lipids, which is particularly relevant when combined with isotretinoin, that may also affect lipid parameters) [99,100,101,102].

The main reason for considering JAK inhibition in DCS is that the JAK/STAT pathway acts as a central point for several cytokines involved in follicular occlusion inflammation. This could allow for broader suppression than targeting a single cytokine, especially in cases that are hard to treat. However, there is very little clinical evidence specific to DCS so far, mostly limited to case reports and findings borrowed from studies in HS. This gap between theory and evidence highlights the need for well-designed studies with standardized outcomes, clear safety checks, and molecular data to show that the pathway is actually being affected in the affected tissue.

### Clinical Perspective

JAK inhibitors and other small-molecule targeted therapies remain experimental in DCS and should be reserved for severe, treatment-refractory cases after failure or intolerance of established systemic therapies and biologics. Off-label use should ideally occur in specialty centers with careful risk–benefit assessment, monitoring, and clear documentation of treatment goals (Table 6).

## 9. Other Systemic Treatment Approaches

In addition to antibiotics, retinoids, and biologics, several unconventional systemic therapies have been explored in the management of DCS (Table 7). However, supporting evidence for these approaches remains limited and largely anecdotal.

Zinc supplementation, known for its anti-inflammatory and antioxidant effects and its ability to modulate cytokines such as TNF-α, has produced mixed results. In a retrospective cohort of eight patients, oral zinc did not result in significant clinical improvement [30]. By contrast, two separate case reports described marked benefit, with one patient maintaining remission for five years and another for at least one year [103,104].

Systemic corticosteroids have occasionally been used in cases of severe or refractory inflammation. One report described the effect of long-term prednisone treatment in a patient who experienced resolution of inflammatory activity within four months and complete scalp hair regrowth after one year; maintenance consisted of 5 mg every other day following multiple tapering courses [105]. This suggests that corticosteroids may be useful for inducing rapid control in highly active disease, although their long-term use is limited by safety concerns.

Hormonal modulation with finasteride at a dose of 1 mg daily has been associated with clinical improvement in two of three reported patients, indicating that androgen suppression may be beneficial in selected individuals [77]. Nevertheless, data are sparse, and finasteride should currently be regarded as an experimental adjunct rather than a standard therapy for DCS.

Another unconventional intervention is saireito, a traditional Japanese herbal medicine with reported immunomodulatory properties. In a small case series, two patients with DCS experienced clinical improvement while taking oral saireito, although pharmacodynamic mechanisms and long-term outcomes were not described in detail [106].

Taken together, these non-standard systemic approaches remain best viewed as individualized adjuncts supported by limited, low-level evidence rather than broadly generalizable options. Reported responses are difficult to interpret because of small sample sizes, frequent concomitant therapies, and limited follow-up, and because many of these interventions lack a clearly defined mechanistic rationale within current models of DCS. Their main value at present is hypothesis-generating. Namely, they help identify potentially relevant biological pathways or clinical niches, but they require systematic evaluation with standardized outcomes before routine incorporation into treatment algorithms.

## 10. Surgical Management

Surgical intervention plays an important role in selected patients with DCS, particularly those with chronic, refractory disease characterized by extensive sinus tracts, scarring, and permanent follicular destruction (Table 7) [26,30]. Surgery is typically reserved for individuals who have not responded adequately to medical therapies or who present with localized, structurally damaged areas in which inflammation has subsided but architectural distortion persists [30]. Relevant reports are summarized in Table 7.

### 10.1. Excision, Drainage, and Curettage

Limited excision or deroofing of fluctuant abscesses or sinus tracts may provide rapid symptomatic relief, especially in patients with painful or draining lesions [107]. Curettage following deroofing has been described as a minimally invasive method to remove the epithelialized roof of sinus tracts and help reduce recurrence. Although short-term improvement is common, durable long-term remission depends on adequate removal of all involved tissue and effective medical control of underlying inflammatory activity [107].

### 10.2. Wide Local Excision

For patients with extensive, end-stage disease, wide local excision remains the most definitive surgical option [26]. Subtotal or total scalp excision has produced sustained remission in multiple reports, often with complete resolution of pain, drainage, and recurrent nodules [108]. Reconstruction following wide excision has been performed using split-thickness skin grafts, local flaps, or secondary intention healing, depending on defect size and anatomic location, with cosmetic outcomes varying accordingly [108,109,110]. Overall, durable disease control is generally excellent when all diseased follicles are removed.

### 10.3. Staged or Combined Procedures

Staged excisions or combined approaches, such as serial full-thickness excisions, deroofing followed by secondary procedures, or excision supported by systemic therapy, have been used to minimize morbidity while maintaining disease control [107,111]. Pre-operative medical therapy, including agents such as systemic corticosteroids or retinoids, may help reduce inflammation and intraoperative bleeding, whereas post-operative maintenance with antibiotics or retinoids may reduce the likelihood of recurrence [107,111].

### 10.4. Pediatric Cases

Pediatric-onset DCS is rare, with most cases reported in adolescence rather than early childhood. Consequently, pediatric-specific evidence is limited and largely confined to isolated case reports and small series. Two case reports show that severe pediatric DCS can require aggressive surgical management: from debridement and grafting in a 14-month-old infant (who had no disease recurrence, normal healing, and abnormal hair regrowth) [112], to complete scalp excision with grafting in a 15-year-old (who also healed well without recurrence) [113].

Procedural and surgical treatments seem most helpful for advanced DCS, especially when scarring, sinus tracts, and permanent follicle damage make medical therapy less effective. The lasting results seen after wide excision or staged procedures likely come from physically removing diseased follicles and tunnels that keep inflammation going. Still, most of the current evidence is retrospective and varies in how recurrence and follow-up are defined. Future research should focus on standardizing how surgical outcomes are reported, including recurrence and hair results, and on providing clearer advice about combining surgery with systemic immunomodulation to lower the risk of relapse.

## 11. Photodynamic Therapy

Photodynamic therapy (PDT) has been considered as a potential adjunctive treatment for DCS, particularly in patients with persistent inflammatory activity or an inadequate response to medical therapy (Table 7). PDT utilizes topical 5-aminolevulinic acid (ALA) or related photosensitizers, which are preferentially absorbed in diseased follicles; subsequent light activation generates reactive oxygen species that reduce inflammation and may decrease microbial biofilm burden [114]. Although evidence remains limited to small observational studies and case reports, outcomes have been generally favorable.

The largest prospective study to date evaluated fire needle pretreatment followed by 5% topical ALA-PDT in patients with DCS and demonstrated meaningful clinical improvement, including reductions in pain, drainage, and lesion size [115]. Additional evidence supports the benefit of PDT in syndromic presentations: ALA-iPDT achieved symptomatic improvement in a patient with the FOT, pachyonychia congenita type II, and ankylosing spondylitis, suggesting utility even in complex disease states [116]. Combination regimens have also been reported. In one case, fire needle pretreatment followed by PDT in conjunction with systemic isotretinoin produced marked improvement in refractory disease [117]. An interim analysis of patients treated with fire needle plus 20% ALA-PDT similarly showed reductions in inflammatory nodules and symptomatic burden [118]. Retrospective data further indicate that ALA-PDT used as an adjunctive therapy can alleviate symptoms and decrease recurrence in patients with persistent DCS [119].

PDT has also been incorporated into multidisciplinary surgical management. According to a small case series, PDT may lead to favorable outcomes when applied before or after excisional procedures, including improvements in wound healing and reductions in disease recurrence [120]. These findings suggest the potential role of PDT both as a standalone adjunct and as part of combined therapeutic strategies.

To sum up, PDT is still a new treatment option with limited supporting evidence. Comparing results is challenging because studies use different protocols, disease stages, and ways of measuring outcomes. When improvements are reported, they may be due to both anti-inflammatory and antimicrobial effects, rather than a clear change in the underlying follicular blockage. Future research should use consistent PDT settings and outcome measures, and should determine if PDT adds any benefit when used with systemic therapy in early or intermediate disease.

## 12. Laser Therapy

Laser therapy has attracted increasing interest as a treatment option for DCS because of its ability to target the hair follicle, the central structure involved in disease pathogenesis (Table 7). By inducing selective follicular destruction, laser devices may interrupt the cycle of follicular occlusion, rupture, and secondary inflammation that characterizes DCS [3]. Evidence remains limited to small series and case reports, but several laser modalities have demonstrated promising results.

One of the earliest documented applications involved carbon dioxide laser excision. In a 1989 case report, a patient achieved complete disease clearance within six weeks following the procedure, with no recurrence observed at four months [121]. Subsequent reports have explored less invasive laser hair-removal techniques as a means of reducing follicular activity. The erbium:YAG laser has been used in two male patients who showed clinical improvement after two and four treatment sessions, respectively [122]. Similarly, long-pulsed ruby laser epilation resulted in favorable outcomes in two male and one female patient, although one individual developed significant AEs, including crusting, erosion, and persistent hypopigmentation [123].

Pulsed diode laser therapy has demonstrated benefit in isolated cases. A 35-year-old man who completed four sessions with an 800-nm diode laser experienced marked reduction in hair density within one month, and remained in remission six months later [124]. Among the most commonly reported modalities is the long-pulsed neodymium-doped yttrium aluminum garnet (Nd:YAG) laser. In one series, four men aged 25–40 years underwent three to seven sessions, resulting in reduced drainage and decreased pain associated with active lesions [125].

Laser-based treatments are mainly localized interventions that may help lower inflammation by targeting specific follicular units and improving drainage in certain areas. Still, there is limited and mostly descriptive evidence, with a lot of variation in devices, treatment methods, and follow-up. Future studies should look at which patients benefit most, use consistent ways to measure results, and see if laser treatments can lower the need for systemic therapy or reduce relapses when combined with other treatments.

## 13. Radiation Therapy

Radiation therapy has been used only rarely in the management of DCS, but historical and modern reports provide insight into its potential efficacy in severe, treatment-refractory cases (Table 7). Early experiences primarily involved X-ray therapy, whereas more recent approaches have utilized electron beam radiation, combined electron–photon techniques, and, in isolated cases, brachytherapy.

X-ray radiation was employed in several studies from the 1950s and 1960s, encompassing a total of nine patients treated for recalcitrant DCS. Despite the limitations of historical techniques and concerns regarding long-term safety, all reported cases demonstrated clinical improvement following therapy [7,126,127]. However, X-ray therapy is no longer considered an acceptable option in modern dermatologic practice due to the substantial risks associated with ionizing radiation, including carcinogenesis, chronic atrophy, and impaired wound healing.

Modern radiation modalities have shown favorable outcomes with improved safety profiles. Electron beam therapy, as well as combined electron–photon radiation, has been reported to reduce nodule size, drainage, and inflammation in four treated patients [128]. Acute side effects were mild and self-limited, consisting primarily of scalp erythema, irritation, pruritus, and dryness [128]. Although patient numbers remain small, these findings suggest that modern external-beam radiation may provide symptomatic relief in select individuals.

Brachytherapy has also been explored in a single case involving a 46-year-old man with occipital DCS who experienced substantial improvement after targeted treatment [129]. Brachytherapy delivers high-dose radiation to the affected tissue with minimal exposure of surrounding tissue. However, the evidence for its use in DCS remains limited to this isolated report.

To sum up, radiation therapy has played a unique role in managing DCS, but it should now be reserved for rare, difficult cases where other treatments have failed. Because the supporting evidence is limited and outdated, and newer targeted immunomodulatory options are available, radiation should be seen as a last resort rather than a standard part of current treatment plans. If radiation is considered today, it is important to clearly document the risks and benefits and to monitor patients over the long term.

**Table 7:** Other systemic and procedural therapies for DCS.

Therapy Type	Study Type	Cohort Size/Patient Details	Regimen/Parameters (Dose + Treatment Duration)	Follow-Up Length	Clinical Outcomes	Main Findings/Outcomes	Ref.
Oral zinc (zinc sulfate)	Case report	n = 1 (adult male)	Zinc sulfate 400 mg three times daily; dose reduced by half after ~12 weeks; total treatment duration ~6 months	~5 years	CR = complete clinical healing with sustained remission	Complete healing and sustained remission reported during follow-up	[103]
Oral zinc (zinc sulfate)	Case report	n = 1 (adult patient with DCS and AC)	Zinc sulfate 135 mg three times daily for ~3 months	Not reported	CR = complete clinical resolution	Marked clinical improvement/clearance reported; follow-up duration not reported	[104]
Systemic corticosteroid (prednisone)	Case report	n = 1 (adult male)	Prednisone administered and transitioned to an alternate-day regimen (maintenance 5 mg every other day); total duration not reported	Not reported	Clinical control = reduction/cessation of inflammatory activity while on therapy	Disease control maintained on low-dose alternate-day prednisone; relapse occurred after discontinuation in some reports	[105]
Finasteride	Retrospective cohort/series (multicenter)	n = 3 treated with finasteride within a larger series	Finasteride 1 mg once daily; treatment duration not reported	Not reported	Response = author-reported improvement	Reported as effective in 2/3 patients in the multicenter series; detailed dosing duration and outcome definitions were not provided	[77]
Saireito (Japanese Kampo medicine) ± antibiotic	Case report	n = 1 (adult male)	Saireito 8.1 g/day orally; used in combination with minocycline 100 mg/day in reported summaries; duration not reported	Not reported	Clinical improvement = reduction in inflammatory lesions/drainage	Clinical improvement reported after initiation; granular timing and follow-up were not reported	[106]
Surgery (excision/drainage procedures)	Retrospective cohort	n = 51 (mixed severity); subset underwent abscess drainage and/or surgical excision	Procedural details (extent of excision, number of stages, and perioperative regimen) were not consistently reported in the cohort report	Not reported	Improvement/relapse per chart review	Surgical approaches (drainage/excision) were used in refractory disease; detailed operative parameters were variably reported	[30]
Surgery (wide excision → split-thickness skin graft)	Case series	n = 2 males (ages 27, 30)	Wide excision with split-thickness skin graft (single definitive procedure)	~5 years	Sustained remission = no recurrence during follow-up	All improved without recurrence; one graft infection reported	[109]
Surgery (wide resection → split-thickness skin graft)	Case series	n = 4 males (ages 27–45)	Wide resection followed by split-thickness skin graft (single definitive procedure)	1–4 years	Sustained remission = no recurrence during follow-up	All improved without recurrence during follow-up	[108]
Surgery (staged excisions)	Case series	n = 2 males (ages 20, 37)	Staged excisions performed every 2–3 months (number of stages not reported)	2–3 months	Remission = cessation of drainage with symptomatic/QoL improvement	Remission of drainage and marked quality-of-life improvement reported after staged excisions	[107]
Surgery (staged excisions + porcine xenograft placement)	Case series/technical report	Not reported	Staged full-thickness excisions with porcine xenograft placement; number of stages and intervals not clearly reported in accessible summaries.	Not reported	Clinical response per author report	Reported successful use as an adjunct for extensive disease; detailed dosing/interval parameters require consultation of the full text.	[111]
Surgery (excision + flap/graft reconstruction)	Case report	n = 1 male (age 65)	Excision with reconstruction using free latissimus dorsi flap and meshed split-thickness skin graft	18 months	CR = complete remission	Complete remission at 18 months; partial graft failure and donor-site seroma	[14]
Surgery (debridement/excision for severe disease with osteomyelitis)	Case report	n = 1 (severe disease with osteomyelitis)	Operative debridement/excision	Not reported	Clinical improvement per author report	Surgical management reported for extensive disease complicated by osteomyelitis; detailed operative parameters not reported	[110]
Surgery (pediatric case)	Case report	n = 1 (pediatric patient)	Management details vary	Not reported	Clinical response per author report	Case report describes management in a pediatric patient	[112]
Surgery (wide local excision; pediatric fulminant disease)	Case report	n = 1 male (age 15)	Wide local excision (single procedure)	9 months	Sustained remission = no recurrence during follow-up	Improved without recurrence during follow-up	[113]
Photodynamic therapy (fire micro-needling + 5% topical ALA-PDT)	Prospective trial	n = 12	Fire micro-needling combined with 5% topical ALA-PDT; multiple sessions	Not reported	Improvement, recurrences also recorded	Majority improved after last session; recurrences common within 1–6 months in responders	[115]
ALA-mediated interstitial PDT (ALA-iPDT)	Case report	n = 1 (follicular occlusion tetrad; pachyonychia congenita type II)	Reported as ALA-based PDT; accessible summary reports 10% ALA-PDT every 3–4 weeks for 3 sessions.	5 months	Improvement = reduced pustules/cysts	Reduction in pustules and cysts at 5 months	[116]
Photodynamic therapy (fire needle pre-treatment + 5% ALA-PDT) ± isotretinoin	Case series	n = 3 males (ages 19–43)	5% ALA-PDT every 2 weeks, 4 treatments total; pretreated by fire needle intervention.	6 weeks–4 months; and 1–2 years	Improvement	All improved at early follow-up; no recurrence reported at 1–2 years	[117]
Photodynamic therapy (fire needle pre-treatment + 20% ALA-PDT)	Case series (interim analysis)	n = 6 males (ages 17–31)	20% ALA-PDT with fire-needle pretreatment; three sessions every 10 days	1 year	CR = complete response; PR = partial response	After 3 sessions: CR 50% (3/6) and PR 50% (3/6); 1-year relapse in 1/6	[118]
Photodynamic therapy (20% 5-ALA; 635 nm light)	Case series	n = 9 males (mean age 26.9)	20% 5-ALA; 635 nm laser for 20 min; 1 treatment (n = 7) or 2 treatments (n = 2).	6 months	Clinical improvement at follow-up	At 6 months, 88.9% (8/9) improved	[119]
Surgery combined with photodynamic therapy	Case series	n = 9	Surgical interventions combined with PDT; exact PDT parameters vary	6 months	Improvement	Combination approach reported clinical improvement	[120]
Carbon dioxide laser	Case report	n = 1 male (age 36)	Carbon dioxide laser (focused mode; power density reported as 31,830 W/cm^2^).	4 months (reported)	CR = complete healing with no recurrence at follow-up	Complete healing by 6 weeks; no recurrence at 4 months	[121]
2940-nm multifractional Er:YAG laser	Case series	n = 2 males (ages 20, 24)	Er:YAG laser once monthly; 2 sessions (n = 1) or 4 sessions (n = 1).	2–4 months	Improvement = reduced lesion count/regression ± hair regrowth	Both improved with lesion regression and partial hair regrowth during treatment course	[122]
Laser-assisted hair removal (ruby laser)	Case series	n = 3 (2 males, 1 female; ages 23–35)	Long-pulsed ruby laser hair removal every 6 weeks; 3–5 treatment sessions	8–10 months	Clinical improvement.	All improved; one patient had persistent hypopigmentation after superficial crusting/erosion	[123]
800-nm pulsed-diode laser	Case report	n = 1 male (age 35)	800-nm pulsed-diode laser every 4 weeks; 4 treatment sessions	6 months	Disease quiescence = absence of active inflammatory lesions	Significant epilation at 1 month; disease quiescent at 6 months (no hair regrowth reported)	[124]
Long-pulsed Nd:YAG laser	Prospective cohort	n = 4 males (ages 25–40)	Long-pulsed Nd:YAG laser; 3–7 treatments performed monthly	1 year	Improvement = decreased drainage/tenderness ± hair regrowth	All improved at 1 year with decreased drainage and tenderness; partial hair regrowth in most patients	[125]
External beam radiation therapy	Prospective trial	n = 4 males (ages 27–42)	External beam radiation therapy (electron beam radiation or combination of electrons and photons) delivered 5 days/week	4–13 years	Sustained response = improvement with no relapse during follow-up	All improved without relapse; complete epilation occurred during or after treatment; scalp irritation/erythema/xeroderma/pruritus	[128]
Superficial brachytherapy	Case report	n = 1 male (age 46)	Superficial brachytherapy: 10 Gy delivered as a fraction of 4.	11 weeks	Clinical improvement with no recurrence at follow-up	Improved at 4 weeks; no recurrence at 11 weeks	[129]
Historical X-ray epilation/radiotherapy	Case series/case reports	Multiple cases	Historical radiotherapy protocols were variably reported; detailed dose/fractionation not consistently reported	Not reported	Clinical improvement	Reports describe improvement associated with epilation; details vary across historical reports	[7,126,127]

Abbreviations: AC, acne conglobate; ALA-PDT, 5-aminolevulinic acid-photodynamic therapy; ALA-iPDT, 5-aminolevulinic acid-mediated interstitial photodynamic therapy; CR, complete response; Er:YAG, Erbium-doped Yttrium Aluminum Garnet; Nd:YAG, Neodymium-doped Yttrium Aluminum Garnet; PR, partial response; QoL, quality of life.

## 14. A Practical Treatment Approach to DCS 

**Figure 2:**
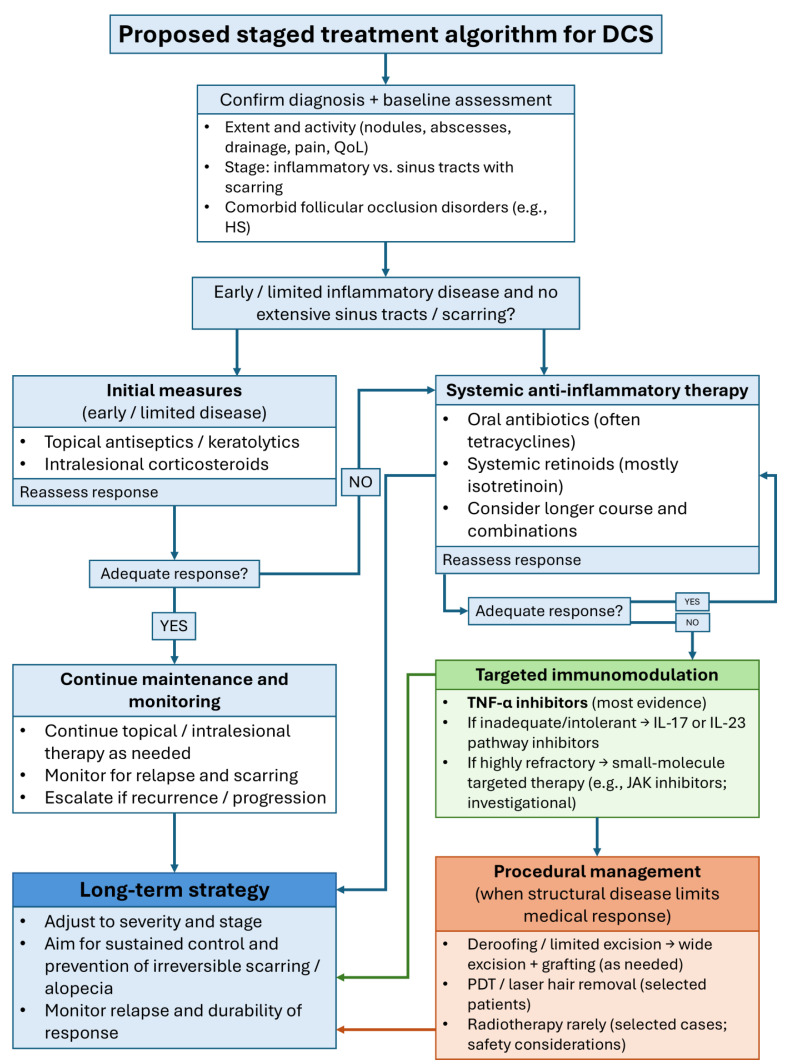
Proposed stepwise treatment algorithm for DCS. This flowchart outlines the step-by-step treatment approach described in Section 14. Early or mild cases focus on controlling inflammation and preventing further follicle damage with topical or injected treatments. If inflammation continues, treatment moves to systemic antibiotics and retinoids. For cases that do not respond or are more complex, targeted immunomodulatory therapies may be considered, such as TNF-α inhibitors, IL-17/IL-23 pathway inhibitors, or JAK inhibitors. When sinus tracts and permanent scarring reduce the effectiveness of medical treatments, procedures like deroofing, excision, laser hair removal, or photodynamic therapy are recommended. This algorithm brings together current evidence to help guide clinical decisions, but it is not meant to replace formal guidelines. Abbreviations: DCS, dissecting cellulitis of the scalp; HS, hidradenitis suppurative; IL, interleukin; JAK, Janus kinase; PDT, Photodynamic therapy; QoL, quality of life; TNF-α, tumor necrosis factor alpha.

Since there are no widely accepted clinical practice guidelines for DCS, the stepwise approach we suggest combines the published evidence on treating DCS and similar follicular occlusion disorders, which are reviewed here (Figure 2).

The management of DCS requires an individualized, phased therapeutic strategy that reflects the chronicity of the disease, its variable inflammatory activity, and the frequent coexistence of other follicular occlusion disorders. In most patients, treatment begins with measures aimed at controlling acute inflammation and preventing further follicular disruption. Topical antiseptics, keratolytics, and intralesional corticosteroids are commonly introduced at the outset, particularly in patients with early or limited disease, although their effects are generally modest and transient.

Systemic therapy becomes necessary for the majority of patients, especially those with persistent nodules, draining sinuses, or progressive scarring. Oral antibiotics, most often tetracyclines, are traditionally used as first-line systemic agents because of their anti-inflammatory and antimicrobial activity. While they frequently improve tenderness and drainage, their benefits are often incomplete and rarely durable after discontinuation. For this reason, systemic retinoids, particularly isotretinoin, remain central to medical management. When used for DCS, isotretinoin is typically administered at relatively higher doses (often in the range of 0.5–1 mg/kg/day) to achieve meaningful disease control, with dose adjustments based on tolerability and comorbidities. They are the most consistently effective agents for achieving disease control and reducing follicular occlusion, although relapse is common once treatment is stopped. Longer courses, maintenance dosing, and combination approaches may be necessary to maintain remission.

Patients with refractory disease, extensive sinus tract formation, or associated HS increasingly benefit from biologic therapy. TNF-α inhibitors such as adalimumab and infliximab represent the most extensively documented biologics in DCS and can produce substantial clinical improvement, particularly in patients with syndromic manifestations. Loss of response, intolerance, or inadequate control may warrant transition to IL-17 or IL-23 pathway inhibitors, which have shown encouraging results in patients with recalcitrant or multisite follicular occlusion disorders. More recently, targeted small-molecule agents, including JAK inhibitors, have emerged as potential options in highly resistant cases, supported by early DCS case reports and a growing evidence base in HS and scarring alopecias. Although their use remains off-label and investigational, they may offer benefit when biologic agents are insufficient.

Adjunctive systemic therapies (including zinc supplementation, short courses of systemic corticosteroids, hormonal modulation with finasteride, and traditional immunomodulatory agents such as saireito) may be incorporated on a case-by-case basis, typically as supplementary rather than primary treatments. These approaches may alleviate symptoms or support other systemic therapies but have limited evidence for long-term disease control.

Procedural intervention becomes appropriate when irreversible follicular destruction or complex sinus tract formation limits the effectiveness of medical therapy. Depending on the severity and extent of disease, surgical approaches range from deroofing and limited excision to wide local excision with grafting. Surgery can provide durable remission when all affected follicles are removed, although the effects and recovery time are variable. PDT and laser hair removal are less invasive therapeutic options that may lead to symptomatic relief and may be recommended to patients with stubborn inflammation or those who cannot tolerate systemic treatments. Radiotherapy has historically been used to achieve clinical improvement, but it is nowadays rarely used due to long-term safety concerns. However, modern electron-based techniques have been successfully used in rare instances.

Taken together, the management of DCS is best approached as a stepwise progression from topical and systemic anti-inflammatory measures to retinoids, targeted biologic or small-molecule therapies, and, when necessary, procedural interventions. Treatment should be adjusted to disease severity, progression, and comorbid follicular occlusion disorders. The emphasis should be on early recognition, sustained disease control, and prevention of permanent scarring.

### Future Research Priorities: Promising Targets, Trial Design, and Biomarkers

Current data show that TNF-α inhibitors are still the most well-supported targeted treatment for DCS, based on the largest patient groups and consistent real-world results in difficult and syndromic cases. The mechanistic framework in Section 3 and Table 1 also makes a strong case for therapies that block Th17-related inflammation. Because of this, IL-17 and IL-23 pathway inhibitors are promising new options, especially for patients who do not respond to or cannot use TNF-α blockers. JAK inhibitors also deserve more study, since the JAK/STAT pathway is a key point for several cytokine signals involved in follicular occlusion disorders, and early reports from DCS cases and related conditions like hidradenitis suppurativa are encouraging. Future research in DCS should focus on comparing these targeted treatments, while also considering disease stage and other health conditions that may affect how patients respond.

When designing clinical trials for DCS, practical methods are needed because the disease is rare. To enroll enough patients, researchers will need to work together across multiple centers and countries. Trials should use a standard way to separate mostly inflammatory disease from more advanced cases with sinus tracts and scarring, since these types may respond differently to treatment. Studies should also report outcomes in a consistent way, including both measures of inflammation (like lesion counts, drainage, pain, and quality of life) and, when possible, objective checks of tract burden and scarring. This is important because advanced structural disease may not improve with medicine alone. Follow-up should last long enough to see if results last and to track relapses, since DCS often comes back after stopping treatment.

Developing biomarkers is a key way to improve how we choose and order treatments. Future research should use both clinical and molecular samples, following the pathway-to-therapy approach in Table 1. This includes examining markers in tissue or blood that indicate TNF-driven inflammation, Th17 activity, and JAK/STAT signaling. Adding objective measures of disease structure, such as sinus tract burden, can help distinguish patients with predominantly active inflammation from those with mostly permanent structural damage. This can make it easier to understand treatment responses. While there are no proven predictive biomarkers for DCS yet, well-organized studies that combine standard outcomes with targeted biomarker collection are the best way to predict responses and guide therapy choices.

## 15. Conclusions

DCS is a chronic follicular occlusion disorder in which follicular rupture initiates innate immune activation and a cytokine network that overlaps with other follicular occlusion conditions, including hidradenitis suppurativa. This overlap supports the use of both treatments that target keratinization and those that modulate the immune response. In practice, the best approach is to start by controlling follicular occlusion and inflammation, usually with systemic retinoids and anti-inflammatory antibiotics. Most patients require combination therapy over an extended period, and maintenance strategies are often necessary to sustain disease control and limit progressive scarring. If the disease escalates, targeted immunomodulation may be needed. Among advanced treatments, TNF-α inhibitors have the most cumulative clinical evidence for DCS, while IL-17/IL-23 inhibitors and JAK inhibitors represent emerging strategies for refractory cases based on cytokine-pathway targeting. Procedures such as surgery, laser hair removal, or photodynamic therapy remain important when sinus tracts or permanent scarring reduce the effectiveness of medical treatments. Since most current evidence comes from case reports and small studies with mixed results, prospective studies with standardized endpoints and biomarker-informed treatment selection are needed to improve remission durability and prevent irreversible alopecia.

## Figures and Tables

**Table 1:** Molecular pathways implicated in DCS and corresponding therapeutic targets.

Molecular/Process Axis	Key Mediators	Mechanistic Role in DCS	Therapy	Refs.
Follicular hyperkeratosis and follicular occlusion	Keratinization, infundibular hyperkeratosis, follicular rupture-associated DAMPs	Follicular occlusion, followed by progressive dilation and rupture, results in the release of keratin, sebum, and microbial components into the dermis. This process triggers a robust innate inflammatory response, leading to the formation of abscesses and sinus tracts.	Systemic retinoids, such as isotretinoin, and keratolytic or follicle-targeting procedures, including laser hair removal, are employed to decrease follicular occlusion and subsequent inflammation.	[1,8,9,10,28,30,38,39,40]
TNF-α-driven inflammatory amplification	TNF-α, NF-κB-dependent inflammatory gene induction	TNF-α induces keratinocyte activation, upregulates endothelial adhesion molecule expression, and facilitates leukocyte recruitment, thereby sustaining the chronic neutrophil-dominated inflammation characteristic of follicular occlusion disorders.	TNF-α inhibitors, such as adalimumab, infliximab, and certolizumab pegol, are administered off-label in cases of refractory or syndromic DCS to disrupt TNF-mediated inflammatory pathways.	[28,29,33,37,47,48]
IL-17 axis (Th17 effector signaling)	IL-17, neutrophil chemokines, antimicrobial peptides	IL-17 signaling promotes neutrophil chemotaxis and stimulates epithelial cells to produce pro-inflammatory mediators and matrix metalloproteinases, thereby sustaining tissue injury and facilitating tract formation.	IL-17A blockade, such as secukinumab or ixekizumab in selected complex cases, may reduce neutrophil-driven inflammation and has demonstrated promising clinical responses in published case reports and series.	[4,19,21,37,54,55]
IL-23 pathway (Th17 maintenance and expansion)	IL-23, IL-12/23, IL-23R, Th17 polarization	IL-23 promotes the survival and expansion of IL-17-producing T cells and maintains a self-reinforcing inflammatory loop, which contributes to the chronicity and severity of follicular occlusion disorders.	IL-23 inhibitors, such as guselkumab, risankizumab, and tildrakizumab, as well as IL-12/23 inhibition with ustekinumab (with mixed results), have been reported in the management of refractory DCS, especially in syndromic phenotypes.	[12,21,22,23,54,55,65]
IL-1β/inflammasome-linked innate immunity	IL-1β, inflammasome activation, neutrophil recruitment	Increased levels of IL-1β have been detected in DCS lesional tissue and serum, which aligns with the presence of a significant innate immune response and subsequent neutrophil recruitment after follicular rupture.	Currently, no IL-1-specific biologic has been established for use in DCS. However, IL-1 blockade, such as with anakinra or other IL-1 pathway antagonists, is considered biologically plausible and investigational, with recommendations primarily extrapolated from HS and autoinflammatory disease literature.	[28,47,48]
JAK–STAT cytokine signal transduction	JAK1/3, STATs; cytokines including IL-6, IL-23, IFN-γ	In follicular occlusion disorders, increased JAK/STAT-related signaling has been observed. JAK-dependent cytokines contribute to sustained inflammation and disruption of regulatory immune mechanisms.	JAK inhibitors, such as upadacitinib and tofacitinib in combination regimens, as well as other agents, are emerging as potential options for highly refractory DCS. Their use is supported by case reports and mechanistic extrapolation from HS trials.	[4,55,56,57,58,59,60]
Fibrosis, scarring, and tissue remodeling (end stage)	Fibroblast activation, extracellular matrix remodeling, TGF-β signaling	Chronic inflammation leads to tissue destruction, which is subsequently followed by granulation tissue formation and fibrotic scarring. Clinically, these processes manifest as cicatricial alopecia and fixed sinus tracts.	Systemic therapies are employed early to control inflammation and prevent irreversible tissue remodeling. In cases of established structural disease, procedural interventions such as deroofing, excision with or without grafting, laser therapy, or photodynamic therapy are utilized to remove or remodel destroyed follicular units.	[25,44,45,46]

Abbreviations: DAMPs, damage-associated molecular patterns; DCS, dissecting cellulitis of the scalp; IFN-γ, interferon-gamma; IL, interleukin; HS, hidradenitis suppurativa; JAK, Janus kinase; NF-κB, nuclear factor kappa B; STAT, signal transducer and activator of transcription; Th17, T helper 17 cell; TGF-β, transforming growth factor beta.

**Table 4:** Anti-TNF biologic therapy in DCS.

Study Type	Cohort Size/Patient Details	Biologic Regimen	Clinical Outcomes	AEs	Ref.
Retrospective cohort	26 patients, refractory DCS; median follow-up 19 months	Infliximab (21 patients), Adalimumab (5 patients)	Significant reductions in nodules, abscesses, PGA, DLQI, pain severity; median satisfaction 7/10	8 discontinued (2 serious AEs: retrobulbar optic neuritis, hepatic cytolysis)	[90]
Case report	19-year-old male with DCS + HS	Adalimumab (40 → 80 mg q2wk)	Resolution of pain/drainage; partial hair regrowth by 3 months; sustained control at 9 months	No serious AEs	[91]
Case report	26-year-old male, DCS + HS + acne	Anti-TNF therapy (unspecified)	Dramatic clinical & QoL improvement over 15 months after isotretinoin/antibiotic failure	Well tolerated	[92]
Case report	Single patient	Human anti-TNF monoclonal antibody (unspecified)	Successful treatment; clinical benefit	Not specified	[93]
Retrospective study	Single patient	Infliximab	No significant clinical improvement over 11.2 months	Not specified	[30]
Case series	2 patients, refractory DCS	Adalimumab after isotretinoin, dapsone, triamcinolone failure	1 complete remission in 3 months; 1 no response after 6 months	None reported	[94]
Case series	2 patients with DCS + HS	Adalimumab (1), Infliximab (1)	Both clinical response: ↓ pain, pruritus, suppuration, acne; sustained 24–32 weeks	Not specified	[59]
Case series	9 patients, recalcitrant DCS	Adalimumab or Infliximab	Improvements in PGA, DLQI, lesions	1 infliximab pt: retrobulbar optic neuritis → discontinued	[18]
Case report	Patient treated for spondylitis + tendonitis	Adalimumab	Sustained stabilization of DCS	Not specified	[95]
Case series	3 patients, refractory to antibiotics/retinoids	Adalimumab	Marked improvement within 8 weeks; 1 relapse post-cessation	Not specified	[29]
Case report	Pregnant woman, co-treated with cephalexin	Certolizumab pegol × 4 months	↓ pain, erythema, discharge; favorable maternal/fetal outcome	None reported	[96]

Abbreviations: AE, adverse effect/event, DCS, dissecting cellulitis of the scalp; DLQI, dermatology life quality index; HS, hidradenitis suppurativa; PGA, physician global assessment; QoL, quality of life; q2wk, every other week; TNF, tumor necrosis factor.

**Table 5:** Case reports on the successful treatment of DCS with secukinumab published to date.

Case	Age/Sex	Disease Presentation	Prior Treatments	Secukinumab Regimen	Clinical Outcomes	AEs/Notes
Schettini et al. (2024)—DCS + HS + AC + pilonidal sinus [20]	24-year-old male	Syndromic: DCS, HS, AC, pilonidal sinus	Isotretinoin, oral antibiotics (tetracycline, rifampicin, and clindamycin), adalimumab (no clinical response)	Standard induction/week 1 (300 mg SC weekly × 5), then every 4 weeks (maintenance)	Gradual improvement, regression of HS (IHS4 score = 9), regression of acne and inflammation, pain and discharge decrease	No side effects reported, but the patient also received intralesional triamcinolone injections
De Bedout et al. (2021)—Isolated DCS [19]	63-year-old male	Isolated, biopsy-confirmed DCS of occipital scalp; ≥4 years duration, scarring alopecia and tender purulent nodules	Oral antibiotics (doxycycline, TMP-SMX, clindamycin, rifampicin), adalimumab (3 months, no improvement), isotretinoin, intralesional triamcinolone, dapsone	Loading phase: 150 mg weekly × 4, then monthly 150 mg; the patient received 8 injections over 3 months (error dosing)	Significant reduction in nodules, abscesses, and suppuration; stabilization of disease activity	Eczematous reaction after therapy initiation (topical treatment)

Abbreviations: AC, acne conglobata; AE, adverse effect/event; DCS, dissecting cellulitis of the scalp; HS, hidradenitis suppurativa; IHS4, International Hidradenitis Suppurativa Severity Score System; TMP-SMX, trimethoprim-sulfamethoxazole.

**Table 6:** JAK inhibitors and other small-molecule targeted therapies reported in DCS.

Study	Patient Characteristics	Disease Duration	Prior Therapies	Small-Molecule Regimen (±Concomitant Therapy)	Treatment Duration	Follow-Up	Clinical Outcomes	Safety/AEs
Islam et al., 2024 [56]	26-year-old male (history of obesity, atopic dermatitis); DCS, posterior scalp	11 months	Topical benzoyl peroxide 10%; TMP–SMX 800/160 mg BID; prednisone 20 mg TID; intralesional triamcinolone 40 mg/mL (multiple visits)	Upadacitinib 15 mg BID added to ongoing regimen	Reported response assessed at 1–2 months (continued thereafter)	2 months reported (continuation planned)	Substantial improvement in pain, draining, and bleeding at 1 month; markedly fewer pustules, smaller sinus tracts, and decreased inflammation without visible drainage at ~2 months	No major side effects reported
Yu et al., 2023 [1]	15-year-old male; DCS	10 months	Minocycline 50 mg BID × 3 months; clindamycin 0.15 g QID × 1 month; I&D surgery (relapse)	Initial: adalimumab 80 mg day 0 then 40 mg q2wk + isotretinoin 30 mg daily × 5 months; Step-up: adalimumab interval extended to Q4W and isotretinoin switched to baricitinib 4 mg daily × 2 months; Maintenance: adalimumab 40 mg every 20 days + baricitinib 4 mg every 3 days × 2 additional months	Baricitinib exposure reported for ~4 months total (2 months daily + 2 months reduced frequency)	9 months total treatment + follow-up	Lesions almost cleared; most inflammatory alopecia patches resolved; improved pain/drainage; hair regrowth reported	Hypertriglyceridemia/hypercholesterolemia during isotretinoin/adalimumab phase; no baricitinib-specific AE reported
Jin et al., 2024 [97]	27-year-old male; refractory DCS	6 years	Topical/systemic corticosteroids; antibiotics; ALA-PDT; isotretinoin; adalimumab; surgery (including incision & drainage)	Abrocitinib 100 mg daily, started after incision and drainage	Clinical remission reported at 4 months	No recurrence reported at 1 year	Achieved remission by 4 months; sustained remission to 1 year	None reported
Qiu et al., 2025 [58]	28-year-old male; severe DCS; BMI 32.8; history of severe acne; smoker	3 years	Oral/topical antibiotics and traditional Chinese medicine (unsuccessful)	Ixekizumab 160 mg loading then 80 mg q2wk + tofacitinib 10 mg/day; doxycycline 200 mg/day × 10 days then isotretinoin 20 mg/day. At 3 months: isotretinoin stopped and ixekizumab reduced to 80 mg Q4W.	10 months total reported	10 months reported	At 3 months: abscesses/exudate markedly regressed with new hair growth; at 10 months: most nodules resolved and DLQI improved from 18 to 6	No serious AEs reported
Al-Mamoori et al., 2025 [98]	29-year-old male; refractory DCS	Not specified	Antibiotics; corticosteroids; isotretinoin (failed)	Tofacitinib 10 mg once daily (off-label)	9 weeks	Remission maintained for 6 months	Marked reduction in inflammation and pain; reduction in pustules and sinus tract activity; clinical remission of active inflammatory lesions by 9 weeks	No reported AE or abnormal labs

Abbreviations: AE, adverse effect/event; ALA-PDT, 5-aminolevulinic acid photodynamic therapy; BID, twice daily; BMI, body mass index; DCS, dissecting cellulitis of the scalp; DLQI, Dermatology Life Quality Index; I&D, incision and drainage; q2wk, every other week; Q4W, every 4 weeks; QID, four times daily; TID, three times daily; TMP–SMX, trimethoprim–sulfamethoxazole.

## Data Availability

No new data were created or analyzed in this study. Data sharing is not applicable to this article.

## Abbreviations

The following abbreviations are used in this manuscript:

AC	Acne conglobata
AE	Adverse effect/event
ALA	5-aminolevulinic acid
ALA-PDT	5-aminolevulinic acid-photodynamic therapy
ALA-iPDT	5-aminolevulinic acid-mediated interstitial photodynamic therapy
BID	Twice daily
BMI	Body mass index
CR	Complete response
DAMPs	Damage-associated molecular patterns
DCS	Dissecting cellulitis of the scalp
DLQI	Dermatology life quality index
Er:YAG	Erbium-doped Yttrium Aluminum Garnet
FOT	Follicular occlusion tetrad
GI	Gastrointestinal
HS	Hidradenitis suppurativa
I&D	Incision and drainage
IFN-γ	Interferon-gamma
IHS4	International Hidradenitis Suppurativa Severity Score System
IL	Interleukin
JAK	Janus kinase
JAK/STAT	Janus Kinase/Signal Transducer and Activator of Transcription
KID	Keratitis–ichthyosis–deafness
MeSH	Medical Subject Headings
Nd:YAG	Neodymium-doped Yttrium Aluminum Garnet
NF-κB	Nuclear factor kappa B
PAMPs	Pathogen-associated molecular patterns
PDT	Photodynamic therapy
PGA	Physician global assessment
PR	Partial response
PS	Pilonidal sinus
QID	Four times daily
QOD	every other day
QoL	Quality of life
q2wk	Every other week
Q4W	Every 4 weeks
RA	Rheumatoid arthritis
STAT	Signal Transducer and Activator of Transcription
TGF-β	Transforming growth factor beta
Th17	T helper 17 cell
TID	Three times daily
TLRs	Toll-like receptors
TMP-SMX	trimethoprim-sulfamethoxazole
TNF	Tumor necrosis factor
TNF-α	Tumor necrosis factor alpha
VTE	Venous thromboembolism
